# Puerarin Relieved Compression-Induced Apoptosis and Mitochondrial Dysfunction in Human Nucleus Pulposus Mesenchymal Stem Cells via the PI3K/Akt Pathway

**DOI:** 10.1155/2020/7126914

**Published:** 2020-01-11

**Authors:** Donghua Huang, Yizhong Peng, Kaige Ma, Xiangcheng Qing, Xiangyu Deng, Zhiliang Li, Zengwu Shao

**Affiliations:** ^1^Department of Orthopaedics, The Second Affiliated Hospital of Zhejiang University School of Medicine, Hangzhou, Zhejiang 310009, China; ^2^Department of Orthopaedics, Union Hospital, Tongji Medical College, Huazhong University of Science and Technology, Wuhan 430022, China

## Abstract

Puerarin (PUR), an 8-C-glucoside of daidzein extracted from Pueraria plants, is closely related to autophagy, reduced reactive oxygen species (ROS) production, and anti-inflammatory effects, but its effects on human nucleus pulposus mesenchymal stem cells (NPMSCs) have not yet been identified. In this study, NPMSCs were cultured in a compression apparatus to simulate the microenvironment of the intervertebral disc under controlled pressure (1.0 MPa), and we found that cell viability was decreased and apoptosis level was gradually increased as compression duration was prolonged. After PUR administration, apoptosis level evaluated by flow cytometry and caspase-3 activity was remitted, and protein levels of Bas as well as cleaved caspase-3 were decreased, while elevated Bcl-2 level was identified. Moreover, ATP production detection, ROS, and JC-1 fluorography as well as quantitative analysis suggested that PUR could attenuate intercellular ROS accumulation and mitochondrial dysfunction. Besides, the rat tail compression model was utilized, which indicated that PUR could restore impaired nucleus pulposus degeneration induced by compression. The PI3K/Akt pathway was identified to be deactivated after compression stimulation by western blot, and PUR could rescue the phosphorylation of Akt, thus reactivating the pathway. The effects of PUR, such as antiapoptosis, cell viability restoration, antioxidation, and mitochondrial maintenance, were all counteracted by application of the PI3K/Akt pathway inhibitor (LY294002). Summarily, PUR could alleviate compression-induced apoptosis and cell death of human NPMSCs in vitro as well as on the rat compression model and maintain intracellular homeostasis by stabilizing mitochondrial membrane potential and attenuating ROS accumulation through activating the PI3K/Akt pathway.

## 1. Introduction

Intervertebral disc degeneration (IDD) is one of the most common pathological disorders around the world, which greatly affects the life quality of patients and imposes enormous financial burden on society [1]. There are many stressors leading to IDD, including genetic susceptibility [2], collagen degradation [3], biomechanical overload, and impaired nucleus pulposus cell (NPC) proliferation [4]. Nucleus pulposus mesenchymal stem cells (NPMSCs), also known as nucleus pulposus (NP) progenitor cells, have similar trilineage differentiation potential to mesenchymal stem cells (MSCs) and were also found to loss cell viability, quantity and properties during IDD [5]. As for its multidirection differentiation ability [6, 7] and tissue specificity, NPMSCs are potentially superior to nonintervertebral disc- (IVD-) derived MSCs for NPC-specific differentiation and might be the potential therapeutic target for IDD. Understanding the effects of unfavorable microenvironment factors on NPMSCs, such as compression, could pave the way for interference and restoration of impaired NP tissues, which is a promising approach to treat IDD [8, 9].

Puerarin (PUR), an 8-C-glucoside of daidzein extracted from Pueraria plants, has been found to be effective in the treatment of many diseases, such as heart failure [10], hypertension [11], cerebrovascular ischemia [12], various cancers [4, 13, 14], Parkinson's disease (PD) [15], Alzheimer's disease (AD) [16], and diabetes as well as diabetic complications [17, 18]. Women after menopause have increased risk of developing IDD, which implies that estrogen reduction is closely associated with IDD [19]. Also, 17*β*-estradiol has been recognized as an inhibitor of IDD by downregulating MMP-3 and MMP-13 and upregulating type II collagen [20]. Similarly, though PUR has not been reported yet in treating IDD, this medicine, as a phytoestrogen [21], is likely to be potentially favorable in suppression of IDD. To be more specific, PUR is greatly associated with modification of autophagy [22], decreased reactive oxygen species (ROS) production [23], and anti-inflammation effects [23]. However, the effects of PUR on apoptosis are different among various kinds of cells. For example, PUR could suppress apoptosis and reduce myocardial injury induced by ischemia [24], while promoting apoptosis in cancer cells [25, 26]. Thus, the specific effects of PUR on the fate of NPMSCs in the development of IDD need to be further clarified.

Several signaling pathways are involved in the pharmacological activity of PUR. PUR inhibited oxidative stress and inflammation which was associated with inactivation of NF-*κ*B signaling [17], and it played a protective role in diabetic liver injury with inhibition on TGF-*β*/Smad signaling. Also, PUR could suppress the p38 MAPK signaling pathway, thus inhibiting vascular smooth muscle cell proliferation [27]. Moreover, the ERK1/2-Runx2 signaling pathway was engaged in the mechanism of PUR in promoting osteogenic differentiation [28]. PUR could attenuate hippocampal neuronal injury through the PI3K/Akt1/GSK-3*β* signaling pathway [29]. In our previous studies, the PI3K/Akt signaling pathway was found to be significantly activated in the protective effect of icariin on human NPCs [30] in unfavorable inflammatory microenvironment. Since PUR can protect the neurocyte also through the PI3K/Akt signaling pathway, it assumes that this mechanism may be adapted to the effects of PUR on NPMSCs.

In this study, we mimic the microenvironment that leads to IDD development by applying a compression apparatus on NPMSCs to study the viability and pathophysiology of the cells. Then, we aimed to find the effects of PUR on impaired NPMSCs and the engagement of the PI3K/Akt signaling pathway.

## 2. Methods

### 2.1. Isolation and Culture of Primary Human NPMSCs

The human NP samples were obtained from five patients who had undergone discectomy for degenerative disc disease, and the details of all patients are shown in Supplementary Table 1. All procedures were approved by the medical ethics committee of Tongji Medical College, Huazhong University of Science and Technology, China. As previously described [31], the NP tissues were isolated from the patients of lumbar disc hernia by a dissecting microscope and washed with phosphate-buffered saline (PBS) and were then dissected and digested for 6 h in 0.25% type II collagenase (Sigma, St. Louis, MO, USA) at 37°C. Samples were then centrifuged twice at 300 × g for 5 min, suspended, and cultured in a complete medium for mesenchymal stem cells (MSCs) (Cyagen, USA) at 37°C in a humidified atmosphere containing 5% CO_2_. Culture medium was changed every 3 days. Cells were digested using 0.25% trypsin-0.02% ethylenediaminetetraacetic acid (EDTA, Sigma) when they reached a confluence of 80–90% and were subcultured in culture flasks at a ratio of 1 : 3. Second generation of NPMSCs was used throughout the following experiments.

### 2.2. Source and Purity of PUR

The main reagent, PUR, was bought from Shanghai Yuanye Biotechnology Co., Ltd. (https://www.shyuanye.com). It was extracted from *Pueraria lobata* and its purity reached 99.9%.

### 2.3. Application of a Compression Apparatus on NPMSCs

A protocol previously established was used, in which NPMSCs were cultured in a stainless-steel pressure vessel to mimic in vivo conditions [4, 32, 33]. The compression apparatus was constructed to withstand up to more than 1.5 MPa pressure. NPMSCs were placed on cell culture plates and exposed to 1.0 MPa pressure as designed below. The pressure vessel was filled with a small quantity of distilled water to maintain adequate humidity and then placed in an incubator at 37°C. Besides, the concentration of CO_2_ was maintained at 5% and monitored by a CO_2_ indicator. Control cells were incubated at 37°C with 5% CO_2_ under 0.1 MPa for 48 hours.

### 2.4. In Vitro Experimental Design

**Figure 1:**
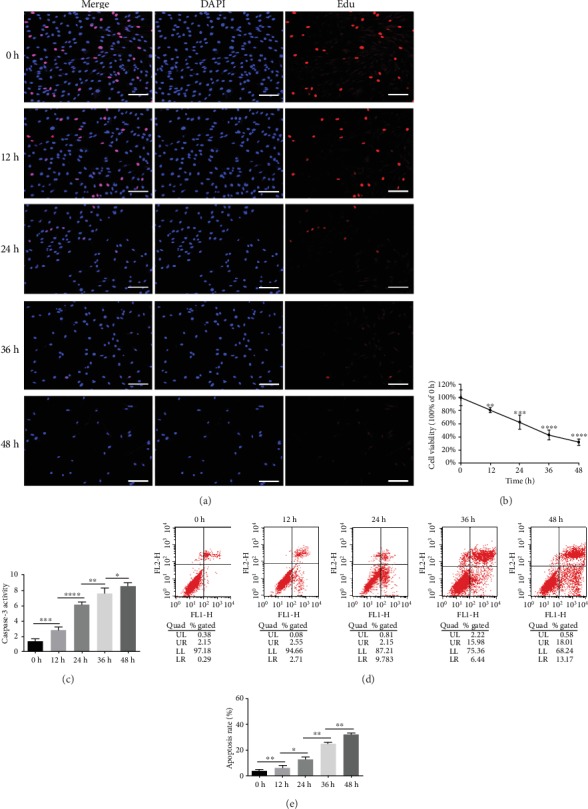
Compression decreased cell viability and induced apoptosis in human NPMSCs. (a) The outcomes of the Edu-staining proliferation assay showed that NPMSC proliferation was progressively impaired by compression (scale bar = 100 *μ*m). (b) The results of the CCK-8 assay suggested that NPMSC cell viability decreased in a compression time-dependent manner. Data were presented as the means ± SD (*n* = 3) (^∗^*P* < 0.05, ^∗∗^*P* < 0.01, ^∗∗∗^*P* < 0.001, and ^∗∗∗∗^*P* < 0.0001 compared with 0 h). (c) Quantitative analysis suggested increasing caspase-3 activity as compression prolonged. (d) Annexin-V/PI staining was performed to determine the apoptosis rate of human NPMSCs by flow cytometry; Annexin-/PI- represents live cells, Annexin+/PI- represents apoptotic cells on early stage, Annexin+/PI+ represents apoptotic cells on late stage, and Annexin-/PI+ represents necrotic cells. (e) Histogram analysis showed the percentage of apoptotic human NPMSCs (Annexin+/PI- plus Annexin+/PI+). Data were presented as the means ± SD (*n* = 3) (^∗^*P* < 0.05, ^∗∗^*P* < 0.01, ^∗∗∗^*P* < 0.001, and ^∗∗∗∗^*P* < 0.0001 between two groups). NPMSCs: nucleus pulposus mesenchymal stem cells; PI: propidium iodide; Edu: 5-ethynyl-2′-deoxyuridine.

To explore the apoptotic promoting effect of compression, NPMSCs were cultured under 1.0 MPa compression for 0 h, 12 h, 24 h, 36 h, and 48 h (Figure 1). To analyze the functions of the PI3K/Akt pathway on the compression-induced apoptosis, ROS accumulation as well as mitochondrial dysfunction, cells were pretreated with 200 *μ*M PUR (Shanghai Yuanye Biotechnology Co., Ltd, https://www.shyuanye.com), 25 *μ*M LY294002 at (an inhibitor of the PI3K/Akt pathway; MCE, China), 200 *μ*M PUR+25 *μ*M LY294002 or the same volume of DMSO (the control) for 24 h and then cultured under 1.0 MPa compression for 48 h.

### 2.5. Cell Viability Assay

After compression of 1.0 MPa for 0, 12, 24, 36, or 48 h, the cellular viability of NPMSCs was examined by a cell counting kit (CCK-8, Dojindo, Japan) according to the manufacturer's instructions. Then, absorbency, indicating cell viability, was detected at 450 nm using a spectrophotometer (ELx808 Absorbance Microplate Reader, Bio-Tek, USA).

For Edu-staining proliferation analysis, NPMSCs were seeded onto a 12-well plate (2 × 10^5^ cells/well). The proliferation of NPMSCs for each treatment group was evaluated in vitro via the EdU DNA Proliferation in Detection kit (RiboBio, Guangzhou, China) according to the protocol. Finally, cells were visualized using an inverted fluorescence microscope (Olympus, Japan).

### 2.6. Western Blot Analysis

NPMSCs were collected and lysed in lysis buffer (Beyotime, Jiangsu, China) containing a mixture of protease inhibitors phenylmethanesulfonyl fluoride (PMSF, Beyotime) and phosphatase inhibitor cocktail I (Sigma, USA). Protein concentrations in cell lysates were determined using an enhanced BCA protein assay kit (Beyotime). Whole lysates were separated using SDS polyacrylamide gel electrophoresis (SDS-PAGE) and transferred onto polyvinylidene fluoride membranes (Amersham Biosciences, USA). Membranes were blocked with 5% bovine serum albumin (BSA, Beyotime) in Tris-buffered saline and Tween 20 (TBST) for 1 h at room temperature. Membranes were then incubated overnight at 4°C with the primary antibodies against Bax (1 : 1000, Abcam, UK), caspase-3 (1 : 1000, Abcam, UK), Bcl-2 (1 : 1000, Abcam, UK), p-Akt (phospho-S473, 1 : 1000, Abcam, UK), Akt (1 : 1000, Abcam, UK), PI3K (1 : 1000, Abcam, UK), p-PI3K (phospho-Tyr524, 1 : 1000, Thermo Fisher, US), and *β*-actin (1 : 5000, Abcam, UK). Membranes were washed three times with 0.1% Tween 20 in TBS for 10 min and incubated with the respective peroxidase-conjugated secondary antibodies for 2 h. After three 10 min washes, the proteins were visualized using an enhanced chemiluminescence (ECL) method according to the manufacturer's instructions (Amersham Biosciences, Piscataway, NJ, USA).

### 2.7. Immunofluorescence

After the indicated treatments, NPMSCs were washed in PBS twice and fixed in 4% paraformaldehyde at room temperature for 15 min. The cells were then blocked for 30 min in 5% bovine serum albumin (BSA) diluted with 0.3% Triton X-100. The cells were incubated with p-AKT (phospho-S473, 1 : 50, Abcam, UK) and PI3K (1 : 50, Abcam, UK) monoclonal antibodies, overnight at 4°C in a dark humidified chamber. After washing, the cells were incubated with a fluorophore-conjugated secondary antibody for 60 min. Then, the cells were counterstained with DAPI in the dark for 5 min. After being washed for 3 times by PBS, stained samples were visualized and photographed using the fluorescence microscope (Olympus, Japan). The integrated optical density (IOD) of p-AKT or PI3K was analyzed in three randomly selected visual fields (per immunofluorescence slice) under high magnification (10 × 40). IOD was measured using the Image-Pro Plus 6.0 analysis system with high resolution. Finally, the relative of IOD value (100% of 0 h) was calculated to make statistical charts.

### 2.8. Apoptotic Assay

Following compression for 48 h, the control and treated NPMSCs underwent trypsinization and centrifugation and were washed with ice-cold PBS and resuspended in 500 *μ*l of 1x binding buffer. 5 *μ*l propidium iodide (PI) and 5 *μ*l Annexin-V (Nanjing Keygen Biotech, Nanjing, China) were then added, and cells were incubated in the dark at room temperature for 15 min. The Annexin and PI double-positive percentage (indicating late apoptosis cells) was measured using flow cytometry (BD LSRII, Becton Dickinson). In addition, NPMSCs were seeded in 6-well culture plates and treated as described above. For each group, cells were rinsed in PBS three times and incubated for 5 min in the dark at room temperature in 5 *μ*mol/l Annexin-V and 5 *μ*mol/l PI. The stained cells were then imaged under a laser scanning confocal microscope (LSM, Zeiss, Germany).

The activity of caspase-3 was measured using a Caspase-3 Activity Detection Kit (Beyotime, China). Briefly, after treatment, both the attached and the floated NPMSCs were totally collected. Next, the supernatant samples were used to evaluate the caspase-3 activity strictly according to the manufacturer's protocols. Finally, caspase-3 activity normalized and the total protein in each group was detected by evaluating the optical density at a wavelength of 405 nm.

### 2.9. Quantitative Real-Time Polymerase Chain Reaction (RT-PCR) Analysis

Total RNA was extracted from human NPMSCs using 1 ml Trizol reagent (Invitrogen, USA) according to the manufacturer's instructions. The isolated RNA was then transcribed into complementary DNA. The primer sequences used for RT-PCR were designed as follows: Bcl-2: forward: 5′-TGAGTTCGGTGGGGTCATGT-3′, reverse: 5′-GGCCGTACAGTTCCACAAAGG-3′; Bax: forward: 5′-CTGACGGCAACTTCAACTGGG-3′, reverse: 5′-TGAGCACTCCCGCCACAAA-3′; caspase-3: forward: 5′-TTGGAACAAATGGACCTG-3′, reverse: 5′-ACAAAGCGACTGGATGAA-3′; *β*-actin: forward: 5′-GTCCACCGCAAATGCTTCTA -3′, reverse: 5′-GTCCACCGCAAATGCTTCTA-3′. Gene expression was quantified through RT-qPCR, using a standard PCR kit and SYBR Green/Fluorescein qPCR Master Mix (5x) (Takara, Japan) on an ABI Prism 7900HT sequence detection system (Applied Biosystems, USA). The amplified products were examined using amplification curve analysis, and all data was analyzed using the 2^-*ΔΔ*CT^ method and normalized to the housekeeping gene *β*-actin.

### 2.10. Transmission Electron Microscopy (TEM)

The ultrastructure of human NPMSCs was examined through TEM. NPMSCs, which had been pretreated by compression or medicine, were collected after trypsinization and centrifugation, then they were washed twice in PBS. Cells were then pelleted for 15 min at 1000 g and supernatant was discarded, before being fixed with 2.5% glutaraldehyde in PBS for 2 h at room temperature. Cells were then postfixed for 2 h with 1% osmium tetroxide, followed by dehydration steps in ethanol and infiltration and embedding in Epon 812. Ultrathin sections were obtained by an ultramicrotome (EM UC7, Leica, Germany) and stained with uranyl acetate and lead citrate and examined with a Tecnai G^2^20TEM (FEI Company, USA).

### 2.11. ROS Assay

The intracellular ROS level was measured using a ROS detection kit (Sigma-Aldrich, St. Louis, MO, USA). The NPMSCs were incubated with 2,7-dichlorofluorescin diacetate (DCFHDA) in the dark at 37°C for 30 min. Then, the cells were washed twice with PBS and measured for ROS production by flow cytometry (BD LSR II, Becton Dickinson), following the manufacturer's instructions. Intracellular ROS levels in each group were also determined by a fluorescence microscope (Olympus, Japan).

### 2.12. Mitochondrial Membrane Depolarization (*Δψm*) Measurement

NPMSCs from each treatment group were collected and resuspended in 1 ml medium containing 10 *μ*g/ml JC-1 (5,5′,6,6′-tetrachloro-1,1′,3,3′-tetraethylbenzimidazolyl-arbocyanine iodide) (Beyotime, China). Then, cells were incubated at 37°C with 5% CO_2_ for 20 minutes and further analyzed by flow cytometry (BD LSRII, Becton Dickinson). The number of detected cells that shift from red to green fluorescence indicates the frequency of cells exhibiting mitochondrial membrane potential (MMP) depolarization. Bandpass filters were 525 ± 25 nm for JC-1 green emission and 610 ± 10 nm for JC-1 red emission. Besides, for the fluorescence staining images, NPMSCs were cultured in 6-well plates and were rinsed in PBS for three times, before being incubated for 30 min in the dark at 37°C in 10 *μ*g/ml JC-1. Then, after being washed with JC-1 Buffer twice, NPMSCs were treated with 1 ml medium for each well and visualized by fluorescence microscope (Olympus, Japan). Intracellular ATP content was measured using the ATP Bioluminescence Assay Kit (Beyotime Biotech) according to the manufacturer's instructions.

### 2.13. Compression and PUR Treatment In Vivo

All animal experiments were carried out with the protocol approved by the Animal Experimentation Committee of Huazhong University of Science and Technology. Nineteen Sprague-Dawley rats (three months old) of 450-500 g in weight were purchased from the Laboratory Animal Center of Huazhong University of Science and Technology (Wuhan, China). To assess the effects of the Ilizarov-type apparatus on different coccygeal discs, 2 rats were sacrificed immediately and the Co7/8 and Co8/9 were collected, and another 2 rats were previously applied with compression stimulation mentioned as follows. Rats were anesthetized with 2% (*w*/*v*) chloral hydrate (40 mg/kg); caudal vertebral bodies (Co7, Co8, and Co9) in the rat tail were located by palpation and counting and confirmed by trial radiography. Then, tails were sterilized by scrubbing with povidone iodine and then affixing the Ilizarov-type apparatus [34]. Two cross 0.7 mm diameter Kirschner wires were inserted by a drill percutaneously into each of the 7th and 9th caudal vertebral bodies, perpendicular to the tail's axis, and fixed on aluminum rings that were connected longitudinally with four threaded rods. After instrumentation, axial loads were applied using four 0.50 N/mm calibrated springs installed over each rod, which were tightened from the distal side to produce a calculated compressive stress of about 1.3 MPa. After the surgery, rats were injected intramuscularly on the quadriceps femoris with penicillin (800,000 U/ml, 0.1 ml) for infection control. After 4 weeks, the rats were sacrificed, and IVDs (Co7/8 and Co8/9) were harvested and prepared for histologic assessment. For another 15 rats, as shown in Supplementary Figure 1, before the application of mechanical loading on rat tails, they were pretreated with saline and PUR by IVD injection [35]. Needles (29 G) were used to puncture the annulus fibrosus layer though the tail skin, in parallel to the end plates. The depth of disc puncture was predetermined based on the dimensions of the annulus fibrosus and the NP, and needles were marked according to the secure depth so as to avoid failed injection. Co7/Co8 discs were injected with 2 *μ*l of saline as the self-control group, while 2 *μ*l of PUR (200 *μ*M) was injected into Co8/Co9 discs as the experimental group. One day after the injection of PUR and saline, they were affixed with the Ilizarov-type apparatus by the method mentioned above. After the operation, the rats were divided into three groups (sham, 2W, and 4W), and each group contained 5 rats. In the 2W and 4W groups, the compression device that imposed 1.3 MPa pressure on rat tails was carried for 2 and 4 weeks, respectively. For the 2W group, when the compression duration reached 2 weeks, the pressure was relieved, but the Ilizarov-type apparatus remained on the tails for another 2 weeks. In the sham group, the compression device was installed without the application of any external mechanical loading. Following the surgery, all rats were housed in standard cages and allowed unrestricted food, water, and activity. Four weeks after the initial surgery, all the rats were sacrificed, and the external apparatus of each rat was uninstalled, then the IVDs (Co7/8 and Co8/9) were harvested and prepared for further assessment. A schematic diagram of in vivo experiment is shown in Supplementary Figure 2.

### 2.14. Histological and Immunohistochemical Analysis

After being washed by PBS for three times, the harvested discs were fixed with buffered formaldehyde (4%, pH 7.4) for 12 h and decalcified in 10% formic acid solution. Then, they were dehydrated by 25%, 50%, 75%, and 95% ethanol, embedded in paraffin, and sectioned at 4 *μ*m. For histological analysis, the sections were stained with hematoxylin/eosin (Beijing Biotopped Science & Technology Co. Ltd., Beijing, China) or Safranin O and Fast Green (Electron Microscopy Sciences, Hatfield, PA, USA) and were visualized with a microscope (Olympus, Japan). A histological grading was applied to determine the cellular and morphological changes in both the annulus fibrosus (AF) and NP (Supplementary Table 2), [36]. The histological sections were assessed by three independent and blinded investigators.

For immunohistochemical analysis, sections were immersed in the permeabilization reagent, 0.5% Triton X-100, for 20 min, and probed with the primary antibody, p-AKT (phospho-S473, 1 : 50, Abcam, UK), at 4°C overnight and washed with PBST for 5 min for three times and incubated with the secondary antibody (1 : 200; Vector, Burlingame, CA, USA) at 37°C for 40 min. Next, the sections were stained with DAPI (Beyotime, China) for 5 min. Then, the images were visualized and photographed with a microscope (Olympus, Japan). The percentage of p-AKT positive cells (p-AKT positive cells/total nuclei) was manually counted to evaluate the effect of PUR on the PI3K/Akt pathway in vivo.

The concrete analysis process of the immunohistochemical slice was as follows: Firstly, only the central slice of Co7/8 (injected by saline, the control) and Co8/9 (injected by PUR) of each rat (five rats per group) were collected. Then, three visual fields (per immunohistochemical slice) under high magnification were randomly selected. Thus, for each treatment group, 15 visual fields (5 immunohistochemical slices∗3 visual fields per slice = 15 fields) were finally included and manually evaluated by three independent investigators. In the situation of a divergence, a consensus was reached by discussion together. Finally, the average percentages of p-Akt positively stained cells were calculated according to the 15 visual fields in each group.

### 2.15. Statistical Analysis

Statistical analysis was conducted using GraphPad Prism 6 software (GraphPad Software Inc., San Diego, CA). All data were obtained from at least three independent experiments and presented as mean ± standard deviation (SD). Multiple sets of data were analyzed by one-way analysis of variance (ANOVA) test, followed by Tukey's post hoc test. Student's *t*-tests were used in the analysis of two-group parameters. Statistical significance was set at *P* < 0.05. All the experiments except the in vivo experiment were repeated independently for three times (biological replicates: *n* = 3). As for in vivo experiments, the number of replicates is fifteen.

## 3. Results

### 3.1. Compression Decreased Cell Viability and Induced Apoptosis in Human NPMSCs

The cells extracted from the degenerated IVD tissues could be effectively induced into osteogenic differentiation, chondrogenic differentiation, and adipogenic differentiation (Supplementary Figure 1A). What is more, the isolated cells showed high levels of MSC-related markers (CD73, CD90, and CD105) and low levels of differential-related markers (CD31 and CD45) (Supplementary Figure 1B). Thus, we successfully isolated NPMSCs, and the cells in passage 2 were used in the present study.

As the time of compression (1.0 MPa) extended, cell viability of NPMSCs was gradually decreased, indicating that the impairment of cell proliferation affected by the compression was time-dependent, and significance was observed in 12 h, 24 h, 36 h, and 48 h groups (Figures 1(a) and 1(b), Supplementary Figure 3). Caspase-3 activity and the percentage of Annexin+/PI+ and Annexin+/PI- cells were increased as the compression prolonged, which suggested the rising apoptotic rate (Figures 1(c)–1(e)).

### 3.2. PI3K/Akt Pathway Was Altered in NPMSCs under the Effects of Compression and PUR

**Figure 2:**
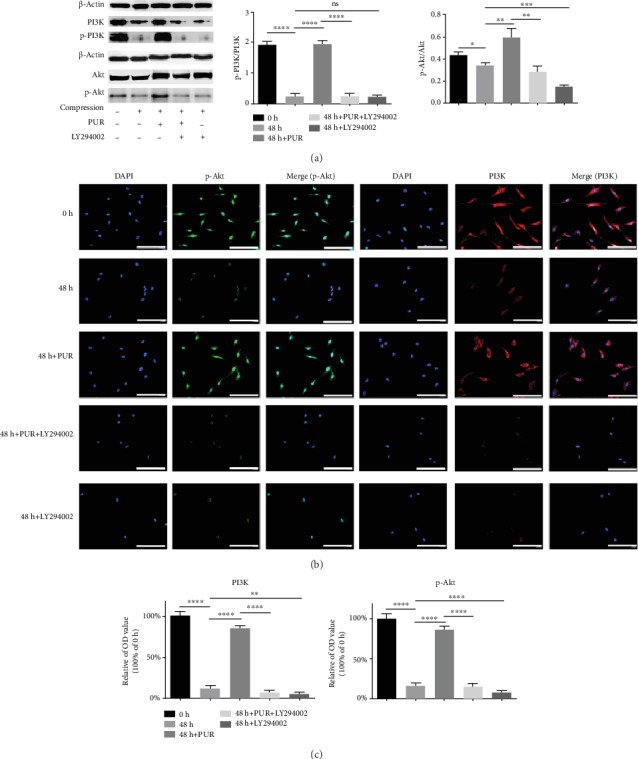
Compression-induced blockage of the PI3K/Akt signaling pathway. (a) Protein levels of PI3K, p-PI3K (phospho-Tyr524), Akt, and p-Akt (phospho-S473) were analyzed by western blotting. Compression: compression loading for 48 h. (b) Immunofluorescence staining of PI3K and p-Akt was observed by a fluorescence microscope (scale bar = 100 *μ*m). (c) Quantitative analysis of PI3K and p-Akt immunofluorescence. Data are presented as mean ± SD (*n* = 3) (ns: no significance, ^∗^*P* < 0.05, ^∗∗^*P* < 0.01, ^∗∗∗^*P* < 0.001, and ^∗∗∗∗^*P* < 0.0001 between two groups). PUR: puerarin.

Inactivation of the PI3K/Akt signaling pathway was observed with decreased p-PI3K (phospho-Tyr524) and p-Akt (phospho-S473), when NPMSCs were under compression of 1.0 MPa for 48 h. However, p-Akt, PI3K, and p-PI3K content was increased with application of PUR, while LY294002, a specific PI3K/Akt pathway inhibitor, can reduce p-PI3K, PI3K, and p-Akt and block the promoting effect of PUR (Figure 2(a)). Similarly, after compression for 48 h, immunofluorescence detected that PI3K and p-Akt were reduced in both the cytoplasm and nucleus. Interestingly, downregulation of PI3K and p-Akt in the cytoplasm was more significant than that in the nucleus. PUR could efficaciously restore compression-induced decrease of PI3K and p-Akt levels in the cytoplasm, while LY294002 administration abrogated this effect radically (Figures 2(b) and 2(c)). Besides, PUR at 200 *μ*M and LY294002 at 25 *μ*M [30] had no effects on cell death and apoptosis, if PUR and LY294002 were pretreated alone without compression (Supplementary Figure 4 and Supplementary Figure 8).

### 3.3. PI3K/Akt Pathway Was Involved in PUR-Related Retardation of Compression-Induced Apoptosis in Human NPMSCs

**Figure 3:**
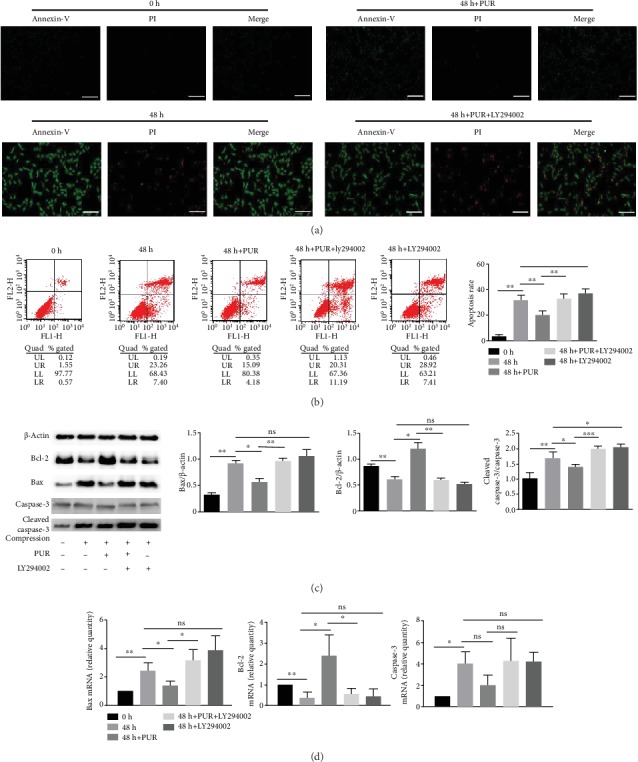
PUR relieved compression-induced apoptosis in human NPMSCs through the PI3K/Akt pathway. (a) Annexin-V/PI staining image indicating the protective role of PUR on compression-induced cell apoptosis through the PI3K/Akt signaling pathway. Green fluorescence represents apoptotic cells in the early stage, whereas red fluorescence represents apoptotic cells in the late stage (scale bars = 50 *μ*m). (b) Apoptosis rate of NPMSCs was determined by flow cytometry; histogram analysis shows the percentage of apoptotic human NPMSCs (Annexin+/PI- plus Annexin+/PI+). Gene expressions (Bcl-2, Bax, and caspase-3) and protein level (Bcl-2, Bax, caspase-3, and cleaved caspase-3) were analyzed by (c) western blotting and (d) real-time PCR. Compression: compression loading for 48 h. Data are presented as means ± SD (*n* = 3) (ns: no significance, ^∗^*P* < 0.05, ^∗∗^ *P* < 0.01, ^∗∗∗^*P* < 0.001, and ^∗∗∗∗^*P* < 0.0001, between two groups). PUR: puerarin; NPMSCs: nucleus pulposus mesenchymal stem cells.

**Figure 4:**
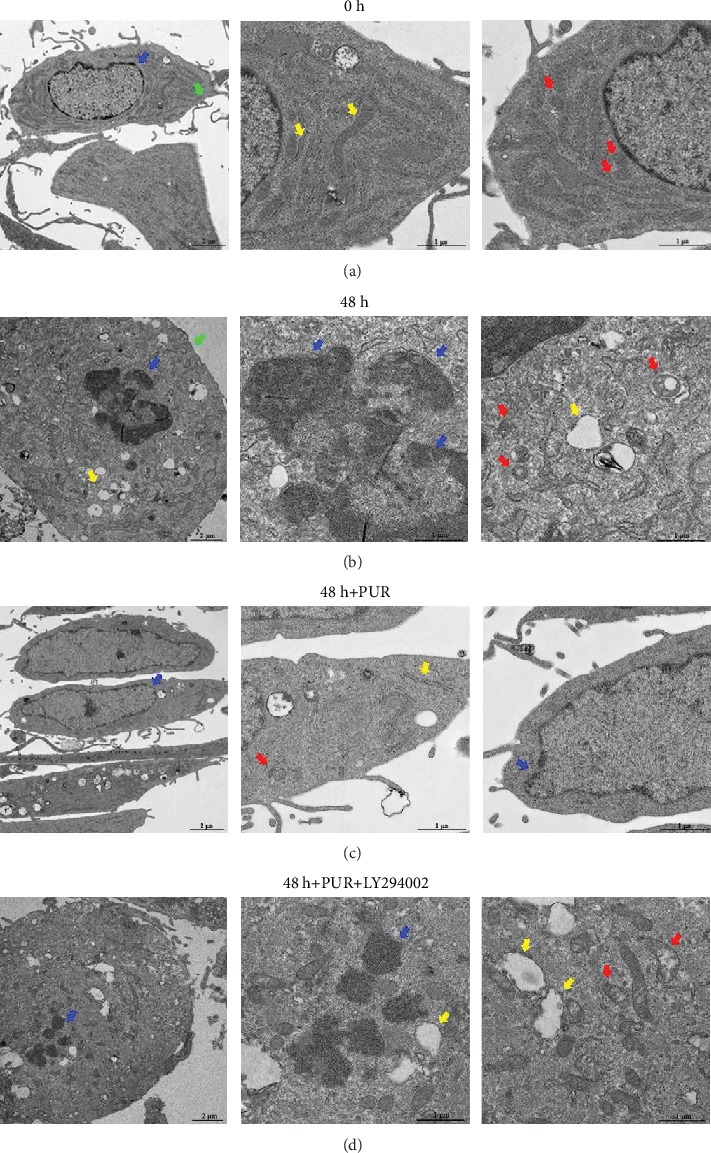
PUR prevented the ultrastructure impairment induced by prolonged compression. (a) The typical cell morphology in the control group (without compression) was detected by TEM. Normal cytomembrane (green), cell nucleus (blue), endoplasmic reticula (yellow), and mitochondria (red) were indicated by arrowheads. (b) Cells demonstrated significant apoptotic morphological changes after 48 h compression. The arrowheads indicate karyopyknosis (blue), vesiculation of endoplasmic reticulum (yellow), and mitochondrial swelling (red). The protuberance of cytomembrane (green) also disappeared. (c) Pretreatment of PUR significantly restored cellular morphology under 48 h compression. Arrowheads indicated relatively normal cell nucleus (blue), mitochondria (red), and endoplasmic reticula (yellow). (d) Apoptotic morphological changes and ultrastructure collapse were observed in NPMSCs after blocking the PI3K/Akt pathway with LY294002. The arrowheads indicate karyopyknosis (blue), vesiculation of endoplasmic reticulum (yellow), and mitochondrial swelling (red) (scale bars = 2 or 1 *μ*m). PUR: puerarin.

Annexin-V/PI fluorescence staining showed a significant increase of cells of the early and late apoptotic phases after 1.0 MPa compression of 48 h. Interestingly, 200 *μ*M PUR effectively alleviated compression-induced apoptosis of NPMSCs on both the early and late apoptotic stages, while the protective effect of PUR was abolished by LY294002 (Figure 3(a)) (the applied concentration of PUR was determined by CCK8, Supplementary Figure 5). Moreover, quantitative analysis revealed that the apoptosis rate of NPMSCs induced by compression was decreased by PUR, and also, this effect could be blocked by LY294002 (Figure 3(b)). The Bax protein level was elevated under 48 h pressure stress, with a decreased Bcl-2 level, indicating the enforcement of apoptosis (Figure 3(c)). Protein level of cleaved caspase-3 was also significantly elevated (Figure 3(c)). With the application of PUR, Bax and cleaved caspase-3 were decreased and Bcl-2 was increased (Figure 3(c)). Consistently, Bax and Bcl-2 transcripts revealed similar patterns (Figure 3(d)). And the effects of PUR were blocked by LY294002. TEM illustrated that the cells' ultrastructure altered significantly after 48 h compression. Karyopyknosis was obvious and there was significant vesiculation of endoplasmic reticulum as well as mitochondrial swelling. Moreover, the protuberance of the cytomembrane disappeared under compression of 48 h, compared to 0 h (Figures 4(a) and 4(b)). Besides, PUR could restore the impaired cellular ultrastructure induced by compression (Figure 4(c)). However, the protective role of PUR could vanish as the apoptotic ultrastructure morphology was significant after additional treatment of LY294002 (Figure 4(d)).

### 3.4. PUR Attenuated Intercellular ROS Accumulation and Mitochondrial Dysfunction in Human NPMSCs under Compression

**Figure 5:**
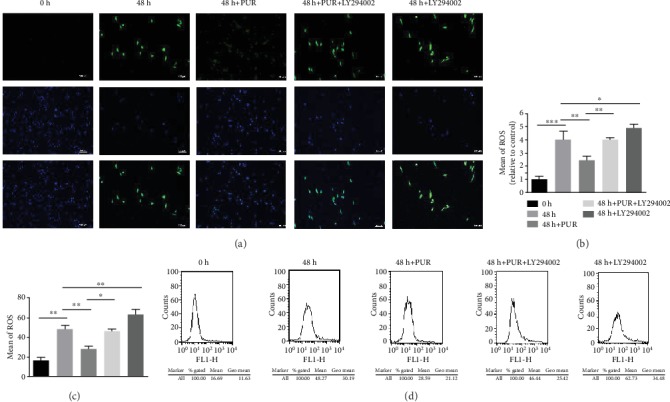
Intercellular ROS accumulation was relieved by PUR in human NPMSCs under compression via the PI3K/Akt pathway. (a) Representative fluorescence imaging of ROS production in human NPMSCs loaded with DCFDA (scale bar = 100 *μ*m). (b) Quantitative analysis of ROS fluorescence staining. (c) Histogram analysis of ROS flow cytometry, which represented the mean fluorescence intensity (the average intercellular ROS accumulation) in NPMSCs in each group. (d) Detection of intercellular ROS production by flow cytometry. Data are presented as the means ± SD (*n* = 3) (^∗^*P* < 0.05, ^∗∗^*P* < 0.01, and ^∗∗∗^*P* < 0.001 between two groups). ROS: reactive oxygen species; PUR: puerarin.

**Figure 6:**
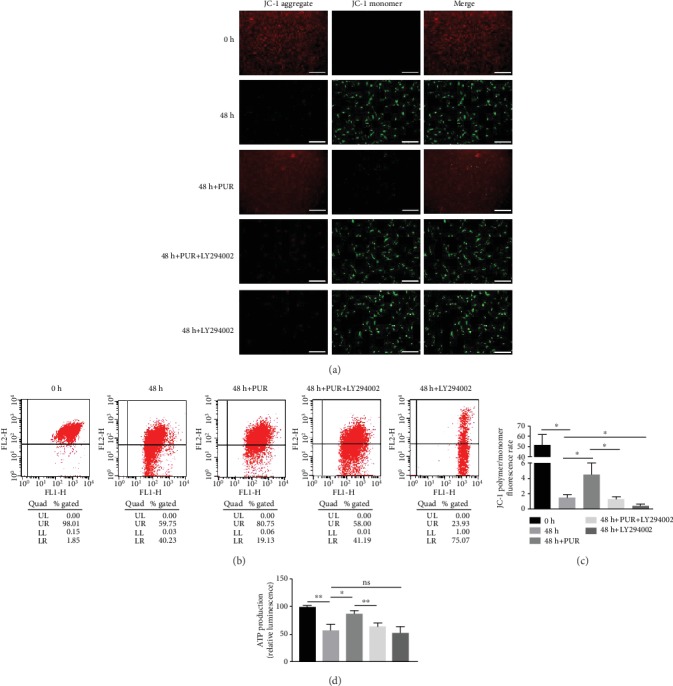
Compression-induced mitochondrial dysfunction was relieved by PUR in human NPMSCs by the activation of the PI3K/Akt pathway. (a) Typical fluorescence photomicrograph of MMP losses was shown by JC-1 fluorescence. Red fluorescence represents the mitochondrial aggregate JC-1 and green fluorescence indicates the monomeric JC-1 (scale bar = 50 *μ*m). (b) Detection of MMP losses using a JC-1 assay kit by flow cytometry. (c) Histogram analysis of the outcomes in (6), which represented level of MMP losses in NPMSCs in each group. (d) ATP production was significantly reduced under compression and PUR promoted ATP production to a certain extent. Data are presented as the means ± SD (*n* = 3) (ns: no significance, ^∗^*P* < 0.05 and ^∗∗^*P* < 0.01 between two groups). PUR: puerarin; NPMSCs: nucleus pulposus mesenchymal stem cells; MMP: mitochondrial membrane potential.

Green fluorescence, indicating ROS production, rose in the 48 h compression group compared to the 0 h control group and decreased by application of PUR (Figures 5(a) and 5(b)). And quantitative analysis by flow cytometry showed that the intercellular generation of ROS was remitted by PUR, and this effect could be disturbed by LY294002 (Figures 5(c) and 5(d)). Furthermore, mitochondrial function was significantly aberrant under compression of 1.0 MPa for 48 h. JC-1 illustrated that the impaired MMP that could be relieved by pretreating PUR (Figures 6(a)–6(c), Supplementary Figure 6). PUR also promoted ATP production, which was significantly impaired by compression, and the favorable effects of PUR was diminished by applying LY294002 (Figure 6(d)). Visualization of swelling mitochondria was shown in Figure 4.

### 3.5. Compression Induced the IDD In Vivo While PUR Relieved It to a Large Extent

**Figure 7:**
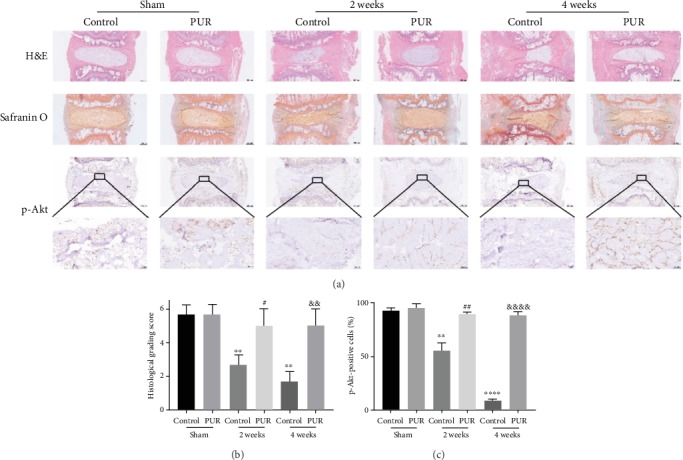
Compression induced the IDD in vivo while PUR relieved it to a large extent. (a) Reactive hematoxylin and eosin (H&E) and Safranin O-fast green (S-O) staining of rat discs from different groups was observed (scale bar = 500 *μ*m). Immunohistochemical detection of p-Akt (phospho-S473) in the NP from different groups were observed (scale bar = 500 *μ*m or 50 *μ*m). IDD: intervertebral disc degeneration. (b) Rat disc degeneration histological grading scores. (c) Statistic evaluation of p-Akt positively stained cells in NP. Data are presented as the means ± SD (*n* = 15) (^∗∗^*P* < 0.01 and ^∗∗∗∗^*P* < 0.0001 compared with the control group of sham, ^#^*P* < 0.05 and ^##^*P* < 0.01 compared with the control group of 2 weeks, and ^&&^*P* < 0.01 and ^&&&&^*P* < 0.0001 compared with the control group of 4 weeks). PUR: puerarin.

As indicated in Supplementary Figure 7, Co7/8 and Co8/9 revealed similar morphology in compression stimulating groups of both 0 week and 4 weeks. Based on that, we considered that it was acceptable to perform PUR injection on Co8/9, while using Co7/8 as the control group with saline injection. Generally speaking, H&E staining showed that the structure was gradually disturbed and the amount of extracellular matrix (ECM) as well as cell number was decreased in control groups, as the compression on the tails extended. Similarly, S-O staining showed a significant reduction of proteoglycans in the control groups of 2 weeks and 4 weeks compression, compared to those of the sham groups. As expected, PUR injection did not significantly aggravate IDD in the sham group. Moreover, H&E staining illustrated that PUR could significantly restore the decreased amount and disturbed distribution of ECM and increase the amount of survival cells, thus alleviating the destructive effect of compression in both the 2W and 4W groups (Figures 7(a) and 7(b)). Furthermore, the percentages of p-Akt-positive cells in the control group of 2W and 4W groups were obviously reduced, while the administration of PUR could increase the protein content of p-Akt of cells in NP tissues (Figure 7(c)).

## 4. Discussion

Due to its adverse impulsion on either individuals or society, IDD has been a highlighted research field, and many factors have been found to contribute to the development of IDD, including age, metabolism, nutrition, and mechanical loading [37]. Among those damaging factors, excessive/abnormal mechanical loading on IVD has been determined to be a primary incentive [38]. Moreover, the underlying mechanism of pressure-induced IDD has now been clarified to a great extent, and many singling pathways have been demonstrated to be involved in the progress, such as TGF-*β*, PI3K/Akt pathway, and p38-MAPK pathway [37, 38].

Recently, IVD progenitor cells, composed of cartilage endplate progenitor cells, annulus fibrosus progenitor cells, and NP progenitor cells [5], were considered to be more efficient for restoring disc height than bone mesenchymal stem cells (BMSCs) [39], thus probably being more promising in IVD regeneration. In order to adequately utilize IVD progenitor cells' differentiation potential, it is vital to fully understand their pathophysiology under unfavorable microenvironment, such as hypoxia and compression, so as to draw a specific therapy to maintain cells' viability and promote the differentiation towards IVD mature cells. In our study, PUR was firstly proven to be protective for human NPMSCs under compression stimulation, and it was effective for restoration of degenerated IVDs in rat models; thus, PUR might be an adjuvant therapy for IVD regeneration.

More specifically, the compression imposed on the human NPMSCs could lead to aggravation of cell apoptosis, resulting in impaired cell viability and anabatic cell death, which is consistent with our previous studies based on rat NPCs [4]. However, there are limited therapies that have been developed based on those findings. PUR, a major bioactive isoflavone extracted from the root of Pueraria lobate [40], has been reported to be effective as a treatment of various pathological conditions. For example, PUR could alleviate liver injury and promote cell proliferation by regulating mTOR signaling pathway [41]. Similarly, PUR, a potential antioxidant, could also relieve cadmium-mediated cytotoxicity in primary rat proximal tubular cells through restoring autophagy and inhibiting Nrf2 pathway [42]. Besides, PUR has also been found to be effective in diabetes mellitus [43] and cardiovascular or neurological disorder [12, 44]. Since PUR is largely related to cell viability restoration and antioxidation, it was hypothesized that PUR might remit compression-induced apoptosis of NPMSCs.

As a result, the administration of PUR could greatly reduce intracellular ROS accumulation and restore mitochondrial dysfunction, which would maintain cellular organelle stability and increase tolerance for compression stimulation, thus rescuing the compression-induced cell death and impaired NPMSC morphology. Though the antioxidant and mitochondrial protective effects of PUR have been proven in myotubes, hyperglycemia-related cardiovascular disease, and subarachnoid hemorrhage [23, 45, 46], this is the first study to identify those effects of PUR on IDD. Moreover, the protective role of PUR on the development of IDD was observed in vivo for the first time. Therefore, PUR might be a potential therapy for human IDD, but the dose and administration of PUR need further clinical exploration to ensure human safety and efficacy. After identifying the protective role of PUR both in vitro and vivo, we then performed further explorations on the latent mechanism.

**Figure 8:**
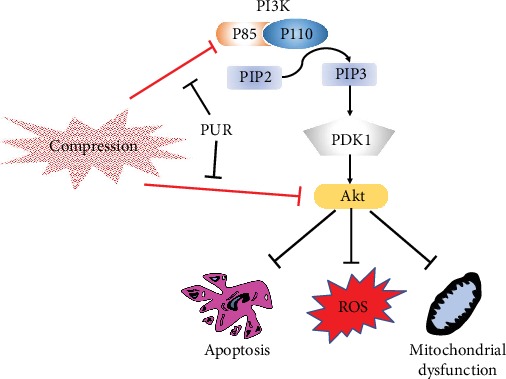
Schematic illustration showing that puerarin could attenuate compression-induced apoptosis, ROS accumulation, and mitochondrial dysfunction of human NPMSCs via the PI3K/Akt pathway. ROS: reactive oxygen species; NPMSCs: nucleus pulposus mesenchymal stem cells.

The PI3K/Akt pathway is a vital intracellular pathway that regulates the cell cycle, which reveals its significant influence on cellular proliferation, aging, cancer development, and apoptosis [47–49]. Compression has been reported to induce NPC apoptosis by downregulating N-cadherin and inhibiting the PI3K/Akt signaling pathway [38]. Activation of the PI3K/Akt signaling pathway might be an approach to rescue cell apoptosis [50]. Interestingly, PUR is an efficient antiapoptosis agent, which can also activate the PI3K/Akt pathway [51]. Specifically, PUR could attenuate cognitive deficits by increasing survival hippocampal CA1 pyramidal neurons [29] and remit daunorubicin-induced apoptosis of H9c2 cells [51] through Akt phosphorylation. Similarly, based on our findings, the PI3K/Akt pathway was inhibited in human NPMSCs under compression, and PUR treatment could reactivate the pathway. Subsequently, the PI3K/Akt pathway inhibitor, LY294002, was applied to block the activation of this pathway after PUR treatment. As a consequence, the effects of PUR, such as antiapoptosis and cell viability restoration, were counteracted. Therefore, we first found that PUR attenuated compression-induced NPMSC apoptosis through the PI3K/Akt pathway. Maintaining mitochondria integrity and preventing cytochrome c and ROS release were engaged in the antiapoptosis effect of PUR [23, 52, 53]. PI3K/Akt deactivation was found to be also linked with mitochondria depolarization [53]. Mitochondrial biogenesis in hepatocytes required functional class 3 PI3K, which controls PPAR*α* transcriptional activity and harmonizes energy demand with mitochondrial content [54]. Inhibition of PI3K was also found to disable the effect of PUR on mitochondrial homeostasis in nonalcoholic fatty liver disease [55]. Consisting with those findings, blocking of PI3K/Akt signaling pathway significantly diminished the effects of PUR on antioxidation and mitochondrial maintenance. Summarily, the protective role of PUR on compression-induced NPMSC cytopathic effects is largely dependent on the activation of the PI3K/Akt pathway (Figure 8). Consisting of the findings in vitro, PUR applied to a rat model was also found to alleviate deformation and structure disorder in vivo with significant activation of p-Akt. However, a few researches reported the anticancerous effect of PUR by inducing apoptosis through inhibiting the PI3K/Akt signaling pathway [26, 56, 57]. Since most of the conflicting studies were performed on cancer cell lines, it was curious that PUR could perform completely contrary effects on apoptosis in different cell lines. Besides, it was also observed by our study that overdose of PUR might cause cytotoxicity, which indicated another possible source of contradictory findings in those studies. Nevertheless, there lacks research that could fully explain the conflicting role of PUR on apoptosis and the PI3K/Akt pathway. Thus, it is suggested that specific molecular interaction between PUR and the PI3K/Akt pathway or its upstream molecules should be further explored.

Recently, there has been another research that evaluated the effects of PUR on lumbar disc herniation (LDH) [58], which highlighted the pain alleviation effects of PUR and pointed out that those effects were spinal ERK-dependent or related to microglia activation. Differently, our study was the first one that investigated the medical effects of PUR on the compression-induced impaired biological behaviors of human NPMSCs and rat IDD development, and we found that those effects were PI3K/Akt pathway-related.

## 5. Conclusions

In conclusion (Figure 8), PUR could alleviate compression-induced apoptosis and cell death of human NPMSCs in vitro as well as on the rat IDD model and maintain intracellular homeostasis by stabilizing MMP and attenuating ROS accumulation through activating the PI3K/Akt pathway. Therefore, PUR might be a potential therapeutic application for intervertebral degeneration disease.

## Data Availability

The data used to support the findings of this study are available from the corresponding author upon request.

## Abbreviations

BMSCs: Bone mesenchymal stem cells
BSA: Bovine serum albumin
DCFHDA: 2,7-Dichlorofluorescin diacetate
ECL: Enhanced chemiluminescence
ECM: Extracellular matrix
EDTA: Ethylene diamine tetra acetic acid
H&E: Hematoxylin/eosin
S-O: Safranin O-fast green
IDD: Intervertebral disc degeneration
IOD: The integrated optical density
IVD: Intervertebral disc
JC-1: 5,5′,6,6′-Tetrachloro-1,1′,3,3′-tetraethylbenzimidazolyl-arbocyanine iodide
MMP: Mitochondrial membrane potential
MSCs: Mesenchymal stem cells
NP: Nucleus pulposus
NPCs: Nucleus pulposus cells
NPMSCs: Nucleus pulposus mesenchymal stem cells
PBS: Phosphate-buffered saline
PI: Propidium iodide
PUR: Puerarin
ROS: Reactive oxygen species
SD: Standard deviation
TEM: Transmission electron microscopy.

## Supplementary Materials

Supplementary Materials Supplementary Table 1: Characteristic details of the patients enrolled in the study. Supplementary Table 2: rat disc degeneration histological grading scale. Supplementary Figure 1: evaluation of NPMSC stemness and cell markers. (A) The ability of NPMSCs to differentiate into osteogenic, chondrogenic, and adipogenic lineages assessed by Alizarin red staining (scale bar = 100 *μ*m), Alcian blue staining (scale bar = 100 *μ*m), and Oil Red O staining (scale bar = 20 *μ*m). (B) Flow cytometric analysis identified the cell surface markers (CD73, CD90, CD105, CD31, and CD45). Supplementary Figure 2: schematic diagram of an in vivo experiment performed on rat models. (A) At the beginning, PUR or saline was injected into the intervertebral discs (Co7/8 and Co8/9). (B) Pressure was loaded on the rat tails at 1 day. (C) After pressure for 28 days, rats were sacrificed and their tails were collected for further investigation. Supplementary Figure 3: quantitative analysis of Edu fluorescence staining. Data were presented as the means ± SD (*n* = 3). (^∗∗^*P* < 0.01, ^∗∗∗^*P* < 0.001, and ^∗∗∗∗^*P* < 0.0001 compared with 0 h). Supplementary Figure 4: the cytotoxicity of PUR and LY294002 on NPMSCs in usage concentration was detected by LDH release assay after 72 h treatment without compression. Data are presented as mean ± SD (*n* = 3). ∗ indicates a significant difference (^∗^*P* < 0.01 and ^∗∗∗^*P* < 0.001 between two groups). Supplementary Figure 5: dose-dependent protective effects of PUR at various points of 1.0 MPa compression duration. The cell viability was detected by cell counting kit (CCK-8) assay. Data were presented as the means ± SD (*n* = 3) Supplementary Figure 6: quantitative analysis of JC-1 fluorescence staining. Data were presented as the means ± SD (*n* = 3) (ns: no significance; ^∗∗^*P* < 0.01 and ^∗∗∗^*P* < 0.001 between two groups). Supplementary Figure 7: reactive hematoxylin and eosin (H&E) and Safranin O-fast green (S-O) staining of rat discs from different groups were observed (scale bar = 500 *μ*m). Co7/8 and Co8/9 illustrated similar destructive performance under 4-week compression. Supplementary Figure 8: the effects of LY294002 on cellular apoptosis in usage concentration (25 *μ*M) were detected by western blot analysis after 72 h treatment without compression. Data are presented as mean ± SD (*n* = 3). ns: no significance between two groups.
