# Supplementary material for: Puerarin Relieved Compression-Induced Apoptosis and Mitochondrial Dysfunction in Human Nucleus Pulposus Mesenchymal Stem Cells via the PI3K/Akt Pathway
Source: Stem Cells Int. 2020 Jan 11;2020:7126914. doi: 10.1155/2020/7126914 (PMC7201526; doi:10.1155/2020/7126914)
Supplement: Supplementary Materials — Supplementary Table 1: Characteristic details of the patients enrolled in the study. Supplementary Table 2: rat disc degeneration histological grading scale. Supplementary Figure 1: evaluation of NPMSC stemness and cell markers. (A) The ability of NPMSCs to differentiate into osteogenic, chondrogenic, and adipogenic lineages assessed by Alizarin red staining (scale bar = 100 μm), Alcian blue staining (scale bar = 100 μm), and Oil Red O staining (scale bar = 20 μm). (B) Flow cytometric analysis identified the cell surface markers (CD73, CD90, CD105, CD31, and CD45). Supplementary Figure 2: schematic diagram of an in vivo experiment performed on rat models. (A) At the beginning, PUR or saline was injected into the intervertebral discs (Co7/8 and Co8/9). (B) Pressure was loaded on the rat tails at 1 day. (C) After pressure for 28 days, rats were sacrificed and their tails were collected for further investigation. Supplementary Figure 3: quantitative analysis of Edu fluorescence staining. Data were presented as the means ± SD (n = 3). (∗∗P < 0.01, ∗∗∗P < 0.001, and ∗∗∗∗P < 0.0001 compared with 0 h). Supplementary Figure 4: the cytotoxicity of PUR and LY294002 on NPMSCs in usage concentration was detected by LDH release assay after 72 h treatment without compression. Data are presented as mean ± SD (n = 3). ∗ indicates a significant difference (∗P < 0.01 and ∗∗∗P < 0.001 between two groups). Supplementary Figure 5: dose-dependent protective effects of PUR at various points of 1.0 MPa compression duration. The cell viability was detected by cell counting kit (CCK-8) assay. Data were presented as the means ± SD (n = 3) Supplementary Figure 6: quantitative analysis of JC-1 fluorescence staining. Data were presented as the means ± SD (n = 3) (ns: no significance; ∗∗P < 0.01 and ∗∗∗P < 0.001 between two groups). Supplementary Figure 7: reactive hematoxylin and eosin (H&E) and Safranin O-fast green (S-O) staining of rat discs from different groups were observed (scale bar = 50 [file 7126914.f1.pdf]

Supplementary Table 1. Characteristics details of the patients enrolled in the study.

Case no.	Age (years)	Sex	Diagnosis	Disc level	Pfirrmann grading
1	68	M	Lumbar disc herniation	L4/5	V
2	59	M	Lumbar disc herniation	L5/S1	V
3	69	F	Lumbar disc herniation	L4/5	V
4	60	F	Lumbar disc herniation	L4/5	IV
5	50	M	Lumbar disc herniation	L4/5	IV

Abbreviation: M, male; F, female.

Supplementary Table 2. Rat disc degeneration histological grading scale

Scores	AF	NP
0	Well-organized fibrous lamellae without ruptured or serpentine fibers	Normal cellularity with large vacuoles and stellar-shaped nucleus consistently dispersed in the nucleus
1	Ruptured or serpentine fibers in less than 30% of the annulus	Slight decrease in the number of cells (<50%) with/without cell clustering
2	Ruptured or serpentine fibers in more than 30% of the annulus with reversed contour	Moderate to severe decrease in the number of cells (>50%) with mostly no vacuolization and occupied by proliferative CNT (<50% of nucleus area)
3	Indistinct and disorganized annulus	Severe replacement by proliferative CNT (>50% of nucleus area) with small area of vacuole cells

AP, annulus fibrosus; NP, nucleus pulposus; CNT, connective tissue.
Scores range from a normal disc (0 point) to severely degenerated disc (6 points).

Supplementary Figure 1. Evaluation of NPMSCs stemness and cell markers. (A) The ability of NPMSCs to differentiate into osteogenic, chondrogenic and adipogenic lineages assessed by Alizarin red staining (Scale bar = 100 μm), Alcian blue staining (Scale bar = 100 μm), and oil red O staining (Scale bar = 20 μm), respectively. (B) Flow cytometric analysis identified the cell surface markers (CD73, CD90, CD105, CD31 and CD45).

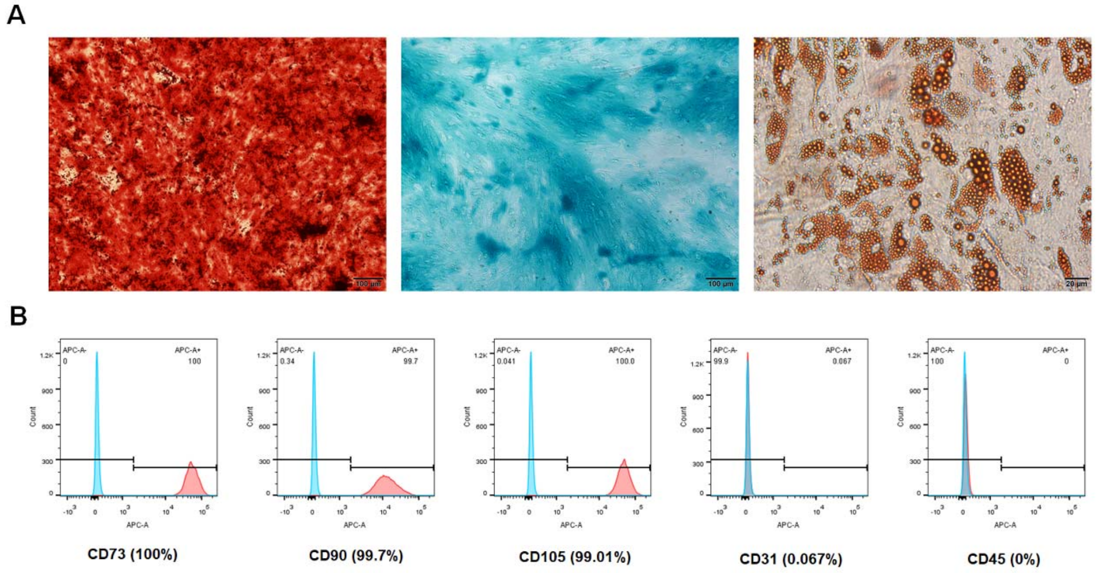

Supplementary Figure 2. Schematic diagram of in vivo experiment performed on rat models. A. at the beginning, PUR or saline was injected into the intervertebral discs (Co7/8 and Co8/9). B. pressure was loaded on the rat tails at day 1. C. after pressure for 28 days, rats were sacrificed and their tails was collected for further investigation.

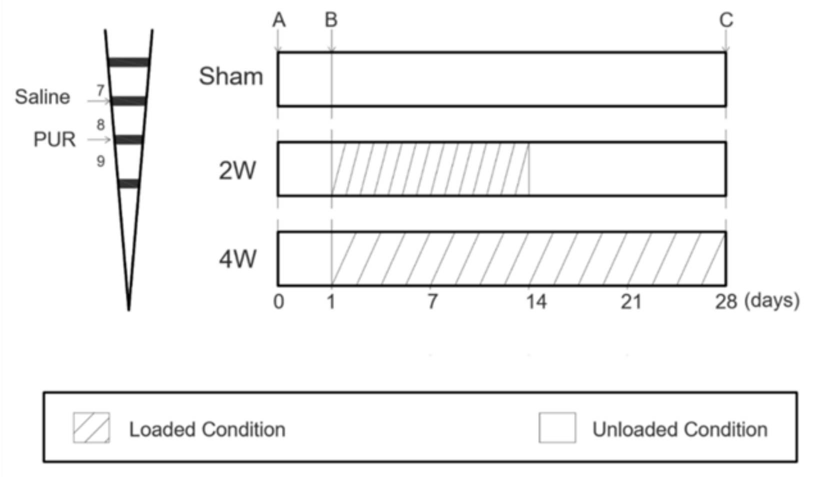

Supplementary Figure 3. Quantitative analysis of Edu fluorescence staining. Data were presented as the means ± SD (n = 3). (***P* < 0.01, ****P* < 0.001, and *****P* < 0.0001 compared with 0h).

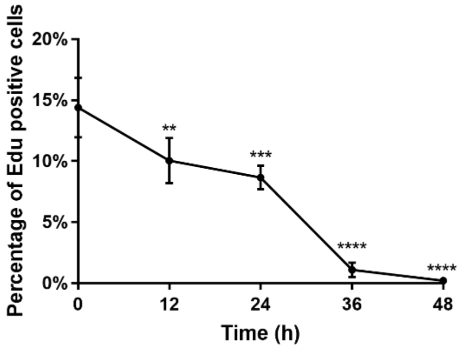

Supplementary Figure 4. The cytotoxicity of PUR and LY294002 on NPMSCs in usage concentration was detected by LDH release assay after 72h treatment without compression. Data are presented as mean ± SD (n=3). *: Indicates a significant difference (**P* < 0.05, ***P* < 0.01) between two groups.

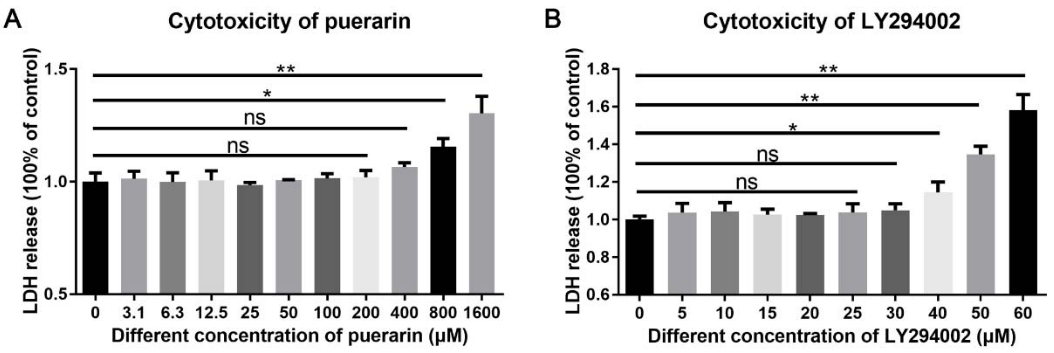

Supplementary Figure 5. Dose-dependent protective effects of PUR at various points of 1.0 MPa compression duration. The cell viability was detected by Cell Counting Kit (CCK-8) assay. Data were presented as the means ± SD (n = 3).

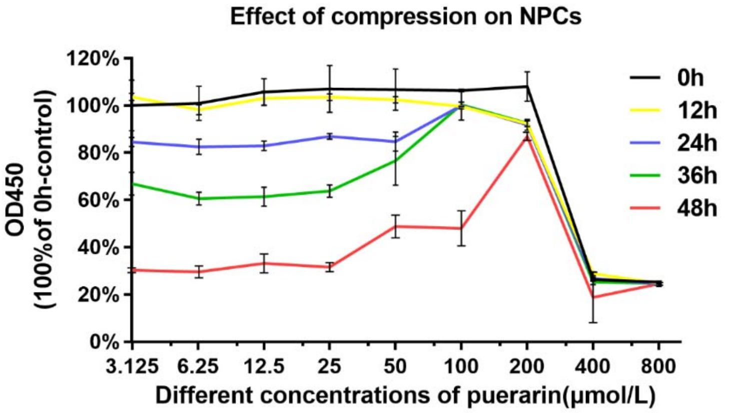

Supplementary Figure 6. Quantitative analysis of JC-1 fluorescence staining. Data were presented as the means ± SD (n = 3). (ns, no significance, $^{**}P < 0.01$, $^{***}P < 0.001$ between two groups).

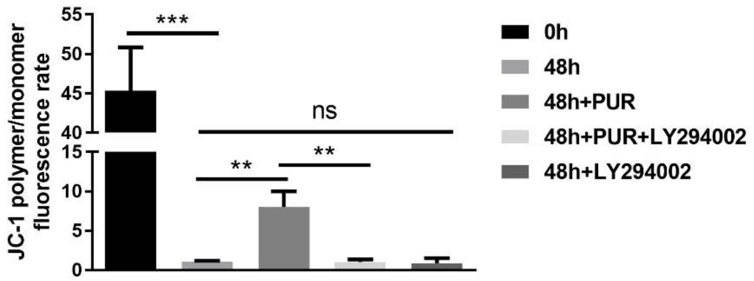

Supplementary Figure 7. Reactive hematoxylin and eosin (H&E) and Safranine O-fast green (S-O) staining of rat discs from different groups was observed (scale bar = 500 μm). Co 7/8 and Co 8/9 illustrated similar destructive performance under 4 weeks compression.

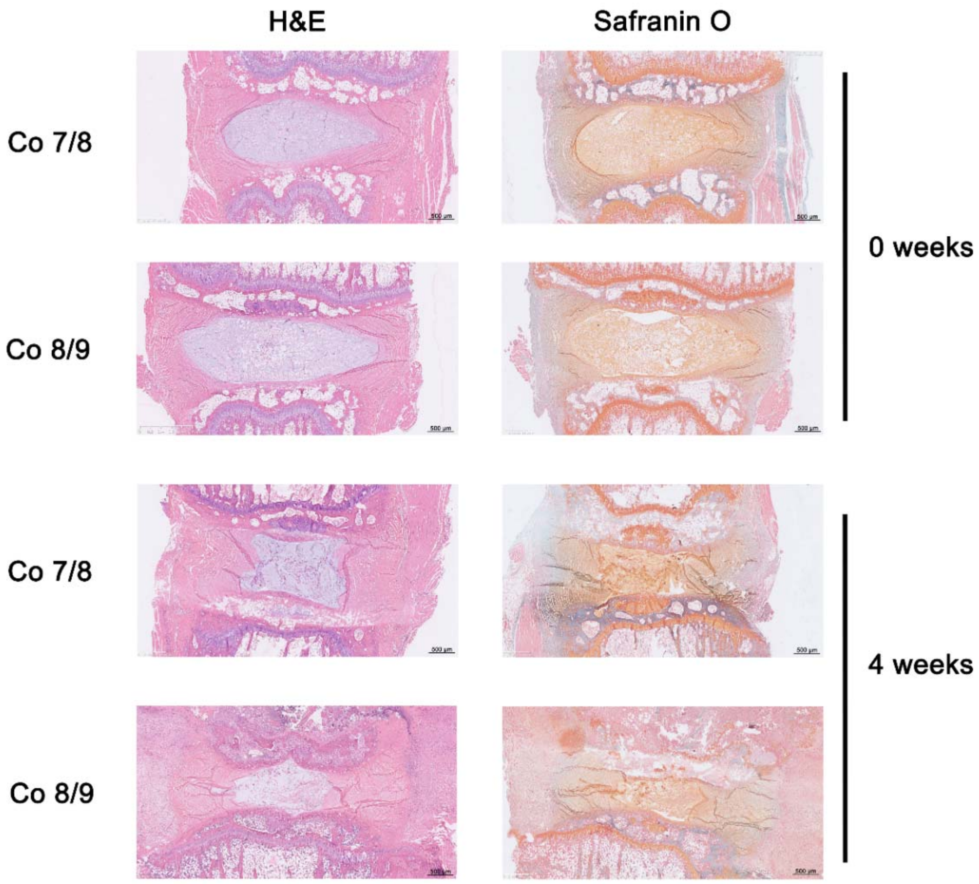

Supplementary Figure 8. The effects of LY294002 on cellular apoptosis in usage concentration (25 μM) were detected by western-blot analysis after 72h treatment without compression. Data are presented as mean ± SD (n = 3). ns, no significance between two groups.

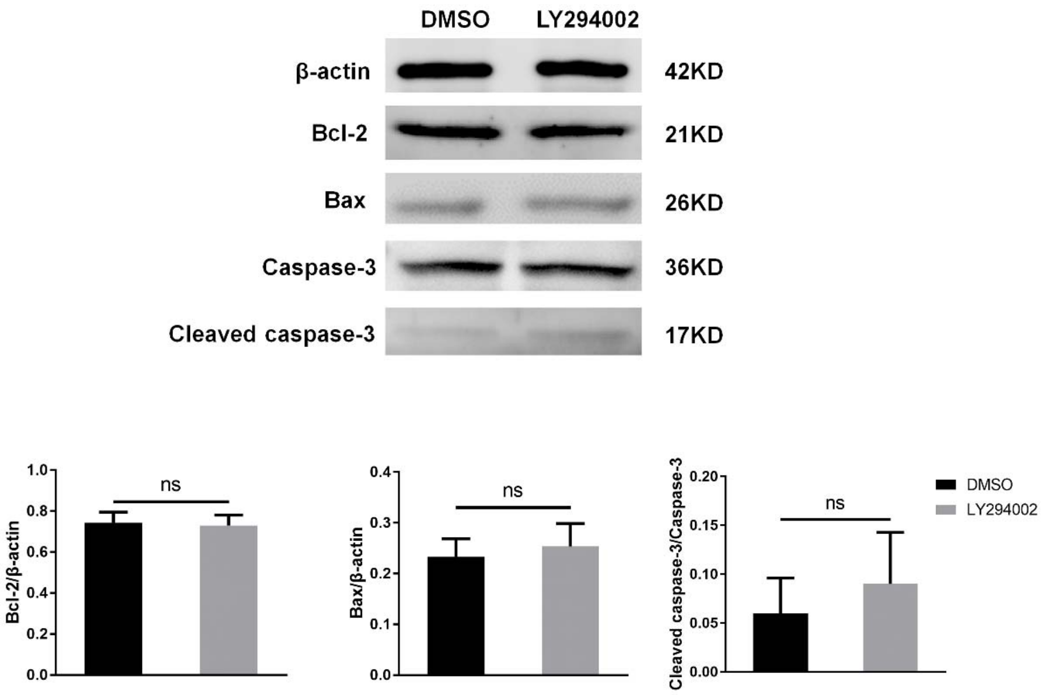
DMSO
LY294002
β-actin
42KD
Bcl-2
21KD
Bax
26KD
Caspase-3
36KD
Cleaved caspase-3
17KD
Bcl-2/β-actin
1.0
0.8
0.6
0.4
0.2
0.0
ns
Bax/β-actin
0.4
0.3
0.2
0.1
0.0
ns
Cleaved caspase-3/Caspase-3
0.20
0.15
0.10
0.05
0.00
ns
DMSO
LY294002
